# Risk factors for early-onset exfoliation syndrome

**DOI:** 10.1038/s41598-022-18738-z

**Published:** 2022-08-30

**Authors:** Do Young Park, Seongyong Jeong, Soon Cheol Cha

**Affiliations:** Department of Ophthalmology, Yeungnam University College of Medicine, Yeungnam University Hospital, Daegu, 42415 Korea

**Keywords:** Glaucoma, Optic nerve diseases

## Abstract

Although exfoliation syndrome (XFS) is an age-related, late-onset disease, early-onset XFS has been reported, and its associated factors remain unknown. In this study, we investigated the clinical features and risk factors of early-onset XFS. The participants were divided into two groups according to age at the time of XFS diagnosis: early-onset (< 60 years) or late-onset (≥ 70 years) group. Among the 302 eyes of 240 patients with XFS, the early-onset group included 41 eyes (14%) of 33 patients, and the late-onset group included 163 eyes (54%) of 126 patients; the mean age was 54.8 ± 5.0 and 76.6 ± 4.9 years, respectively (*p* < 0.001). All eight cases diagnosed with XFS at the earliest age, ranging from 36 to 52 years, underwent trabeculectomy before the diagnosis of XFS. Multivariable logistic regression analysis showed that a history of trabeculectomy (odds ratio [OR] = 11.435, *p* < 0.001), presence of iridectomy (OR = 11.113, *p* < 0.001), and longer axial length (OR = 2.311, *p* = 0.003) were significantly associated with the development of early-onset XFS. Collectively, patients with early-onset XFS were more likely to have undergone trabeculectomy and have more axial myopia compared with those with late-onset XFS. These findings suggest that surgical trauma compromising the blood-aqueous barrier may trigger early manifestation of XFS.

## Introduction

Exfoliation syndrome (XFS) is an age-related systemic disorder characterised by the abnormal deposition of fibrillary materials in the extracellular matrix^1,2^. In the eye, exfoliation materials (XFM) may be deposited in the trabecular meshwork, causing intraocular pressure (IOP) elevation and subsequently leading to development of exfoliation glaucoma^1,3,4^.

Although various risk factors associated with XFS have been identified, the pathogenesis of XFS has not been fully understood^3^. Most notably, specific *LOXL1* alleles have been reported to be strongly associated with XFS^3,4^. However, it remains unclear why risk variants are also common in healthy participants and why their effects are different across ethnicities^3,5^. Besides genetic predisposition, several environmental factors, such as geographic and climatic factors, sunlight exposure, and diet, are known to contribute to the development of XFS, although the precise mechanism remains unclear^3^. Furthermore, based on aqueous humour (AH) analysis from XFS eyes, transforming growth factor beta-1 (TGF-β1), pro-inflammatory cytokines, and oxidative stress have also been suggested to play an important role in the pathogenesis of XFS^6–8^.

Considering that the prevalence of XFS increases with age and that most cases of XFS develop in individuals aged over 60 years^5–9^, increasing age is a definite risk factor for the development of XFS^5,9^. Interestingly, however, XFS may develop at a young age in some cases^10,11^. We believe that investigating the clinical features of patients with early-onset XFS and the associated risk factors can provide insights into the pathogenesis of XFS. Additionally, specifying the risk factors for XFS can help predict the disease-related prognosis of patients. Thus, this study aimed to compare the clinical features of patients with XFS according to disease onset and to identify factors associated with early-onset XFS.

## Methods

We retrospectively analysed the medical records of patients with XFS (with or without glaucoma) who visited the Glaucoma Clinic at Yeungnam University Hospital between January 2000 and October 2015. This study was approved by the Institutional Review Board (IRB) of Yeungnam University Hospital and followed the tenets of the Declaration of Helsinki (IRB No. 2015-01-033). The IRB of Yeungnam University Hospital waived the requirement for informed consent due to the retrospective nature of the study.

XFS was defined as the presence of typical XFM in the anterior segment of the eye. The location of XFM was documented in detail by a glaucoma specialist (S.C.C). In phakic eyes, XFS was diagnosed when the presence of XFM was observed on the surface of the anterior lens capsule and/or the pupillary margin. In pseudophakic eyes, the diagnosis was made when there was a pattern of radial striations on the anterior surface of the intraocular lens and/or scattered dot-like XFM deposits on the posterior capsule and posterior surface of the intraocular lens^12,13^. Suspected cases of XFS, wherein the presence of XFM was observed only at the pupillary border, were excluded from this study.

Glaucoma was defined as characteristic glaucomatous optic disc changes (neuroretinal rim notching or thinning, retinal nerve fibre layer defects or disc haemorrhage, or cup-to-disc ratio asymmetry > 0.2 between the eyes) with corresponding visual field abnormalities. A glaucomatous visual field defect was confirmed when at least two of the following criteria were satisfied: (1) a cluster of three points with a probability of less than 5% on the pattern deviation map in at least one hemifield, including at least one point with a probability of less than 1%, or a cluster of two points with a probability of less than 1%; (2) a glaucoma hemifield test result outside the normal limits; or (3) a pattern standard deviation of 95% outside the normal limits.

Patient data, including age, sex, axial length, refractive error, lOP, presence of glaucoma, use of glaucoma medications, previous history of intraocular surgery or ocular trauma, and accompanying ocular or systemic diseases, were collected from the medical records at the time of diagnosis of XFS. Details on the history of intraocular surgery were collected separately for cataract surgery, trabeculectomy, and Ahmed valve implantation, together with the iridectomy status. Regarding the presence of a systemic disease, information on diabetes, hypertension, cerebrovascular disease, and ischaemic heart disease was collected. The mean IOP was defined as the average IOP value within 3 months, including the time of diagnosis of XFS. Axial length was measured using A-scan ultrasonography (Ultrascan Imaging System; Alcon, Fort Worth, TX, USA). Refractive error values were recorded and converted to spherical equivalents only in patients with phakic eyes.

Statistical analyses were performed using the IBM SPSS software (version 24.0; IBM Corp., Armonk, NY, USA) and the R statistical package version 3.5.3 (R Foundation for Statistical Computing, Vienna, Austria). If the patient had bilateral XFS, both the eyes were examined separately as independent eyes. For comparison, patients were classified into two groups according to age at the time of XFS diagnosis: the early-onset XFS group (< 60 years) and the late-onset XFS group (≥ 70 years). The Mann–Whitney U test was used to compare the mean values of continuous variables between the two subgroups, and the chi-squared test or Fisher’s exact test was performed to compare categorical variables. Univariable and multivariable logistic regression analyses were conducted to identify the factors associated with early-onset XFS. Factors with a *p*-value < 0.1 in the univariable regression analysis were included in the multivariable analysis. In the multivariable regression analysis, two models were used to avoid multicollinearity. Multicollinearity was examined by calculating the variance inflation factors (VIFs), and those with a VIF > 5 were excluded. Statistical significance was set at *P* < 0.05.

## Results

Of the 302 eyes in 240 patients with XFS, 41 eyes of 33 patients diagnosed with XFS under the age of 60 years (early-onset group) and 163 eyes of 126 patients diagnosed with XFS over the age of 70 years (late-onset group) were included in the analysis. All the patients included in this study were Korean. To highlight the differences in the characteristics of patients according to age at the time of XFS diagnosis, 98 eyes of 81 patients diagnosed with XFS between the ages of 60 and 70 years were excluded from the analysis.

The mean age was 54.8 ± 5.0 (median, 57) and 76.6 ± 4.9 (median, 76) years in the early-onset and late-onset groups, respectively (*p* < 0.001). The proportion of men was higher in the early-onset group than in the late-onset group, although the difference was not statistically significant (65.9% vs. 52.2%, *p* = 0.115; Table 1). The proportion of patients diagnosed with XFS in both the eyes did not differ significantly between the early- and late-onset groups. No significant difference was noted in systemic diseases, such as diabetes, hypertension, cerebrovascular disease, and ischaemic heart disease between the two groups (Table 1).

**Table 1:** Comparison of characteristics between patients with early-onset and late-onset XFS.

	Early-onset (n = 41)	Late-onset (n = 163)	*p*-value
Age (years)	54.8 ± 5.0	76.6 ± 4.9	< 0.001*
Sex (male/female)	27/14	85/78	0.161^†^
Diabetes (%)	7 (17.1)	43 (26.4)	0.301^†^
Hypertension (%)	17 (41.5)	84 (51.5)	0.328^†^
Cerebrovascular disease (%)	6 (14.6)	16 (9.8)	0.400^†^
Ischaemic heart disease (%)	4 (9.8)	8 (4.9)	0.243^†^
Retinal vessels occlusion (%)	3 (7.3)	7 (4.3)	0.434^†^
Mean IOP (mmHg)	19.5 ± 10.5	17.7 ± 7.5	0.299*
Presence of glaucoma (%)	34 (82.9%)	108 (66.3%)	0.060^†^
Laterality (bilateral/unilateral)	18/23	74/89	0.863^†^
Usage of glaucoma medications (%)	21 (51.2%)	87 (53.4%)	0.805^†^
Axial length (mm)	24.9 ± 2.8	23.1 ± 1.0	0.013*
Refractive error (diopter)	− 1.43 ± 2.87	0.11 ± 1.70	0.006*
History of intraocular surgery (%)	19 (46.3)	39 (23.9)	**0.008**^**†**^
History of trabeculectomy (%)	13 (31.7)	13 (8.0)	**< 0.001**^**†**^
History of cataract surgery (%)	6 (14.6)	32 (19.6)	0.610^†^
Presence of iridectomy (%)	15 (36.6)	13 (8.0)	**< 0.001**^**†**^

Data are presented as mean ± standard deviation or frequency (%).

IOP, Intraocular pressure; XFS, exfoliation syndrome.

*p-value was calculated using the Mann–Whitney U test.

^†^*p*-value was calculated using the chi-squared test or Fisher’s exact test.

Statistically significant values are shown in bold.

The mean IOP was not different between the two groups (early-onset: 19.5 ± 10.5 vs. late-onset: 17.7 ± 7.5 mmHg, *p* = 0.299). There was no significant difference in the current use of glaucoma medication (Table 1). The axial length was longer in the early-onset than in the late-onset group (24.9 ± 2.8 vs. 23.1 ± 1.0 mm, *p* = 0.013), and refractive error was more myopic in the early-onset than in the late-onset group (− 1.43 ± 2.87 vs. 0.11 ± 1.70, *p* = 0.006; Table 1).

**Figure 1:**
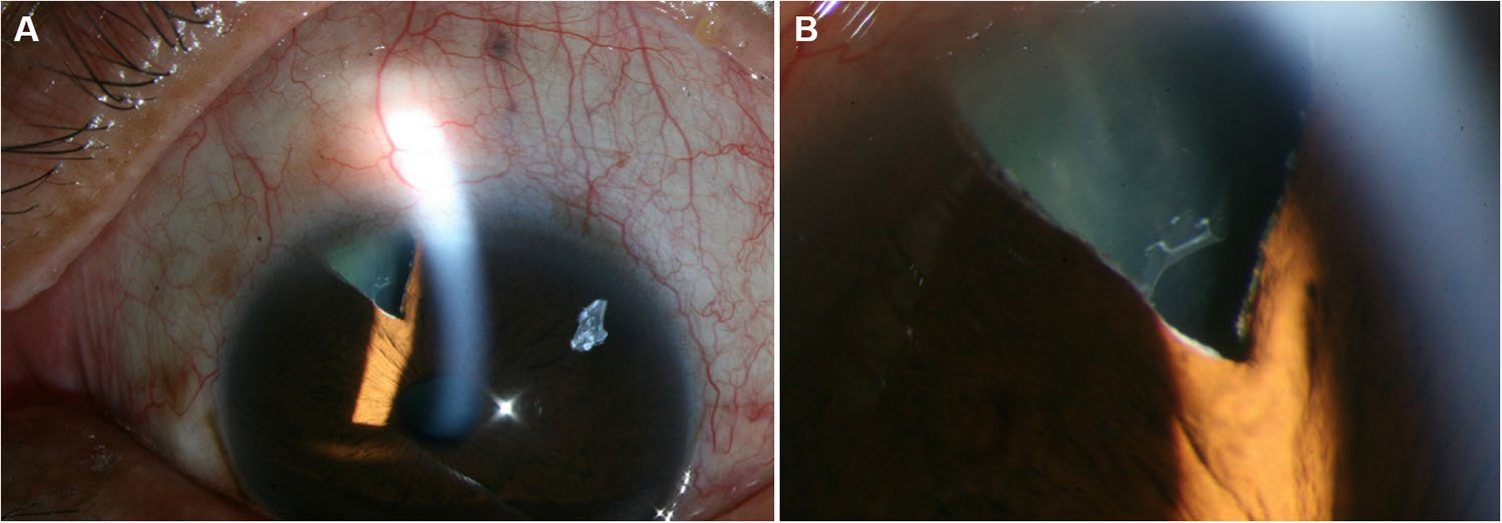
A case of early-onset exfoliation syndrome. The patient was diagnosed with primary open-angle glaucoma (POAG) in both eyes at the age of 51 years and underwent trabeculectomy in both eyes at the age of 54 years. At the age of 57 years, 3 years after surgery, exfoliation material was first noticed in the left eye. Typical exfoliation material is observed on the anterior surface of the lens capsule and at the iridectomy site (**A**). A magnification image of the iridectomy site (**B**).

The proportion of eyes with a history of intraocular surgery was higher (*p* = 0.008) in the early-onset group (19 eyes, 46.3%) than in the late-onset group (39 eyes, 23.9%). When eyes with previous intraocular surgery were analysed separately from those with previous cataract surgery and trabeculectomy, no significant difference was noted in the proportion of eyes with previous cataract surgery between the two groups (six eyes, 14.6% vs. 32 eyes, 19.6%, *p* = 0.610; Table 1). However, the early-onset group had a significantly higher proportion of eyes with previous trabeculectomy than the late-onset group (13 eyes, 31.7% vs. 13 eyes, 8.0%, *p* < 0.001). In particular, the eight eyes diagnosed with XFS at the earliest ages, ranging from 36 to 52 years, among all the eyes examined in this study, had a history of trabeculectomy. Among the 19 eyes with previous surgical history in the early-onset group, 15 eyes were of patients whose medical records existed in our clinic before the surgery and were available sources of longitudinal data. Among them, 12, 2, and 1 eye were treated with trabeculectomy, extracapsular cataract extraction (ECCE), and phacoemulsification, respectively. XFM was not observed in any of the 15 eyes before surgery, and the mean interval from the time of surgery to the manifestation of XFM was 41.3 ± 17.4 (range, 14.8–83.9) months. A representative case from the early-onset group, wherein XFM was observed after trabeculectomy, is presented in Fig. 1.

In the univariable logistic regression analysis, history of intraocular surgery, history of trabeculectomy, presence of iridectomy, long axial length, and presence of glaucoma were significantly associated with early manifestation of XFS. Intraocular surgery was excluded from the multivariable analysis because of multicollinearity (VIFs = 6.78). The multivariable logistic regression analysis showed that a history of trabeculectomy, presence of iridectomy, and long axial length remained significant risk factors associated with the early-onset of XFS (Table 2).

**Table 2:** Factors associated with early-onset XFS using logistic regression analysis.

Variables	Univariable		Multivariable			
			Model 1		Model 2	
	Odds ratio (95% CI)	*p*-value	Odds ratio (95% CI)	*p*-value	Odds ratio (95% CI)	*p*-value
Sex, male	1.770 (0.866–3.617)	0.118				
Diabetes, yes	0.575 (0.237–1.393)	0.220				
Hypertension, yes	0.666 (0.333–1.332)	0.251				
Cerebrovascular disease, yes	1.575 (0.575–4.316)	0.377				
Ischaemic heart disease, yes	2.081 (0.595–7.283)	0.252				
Retinal vessels occlusion, yes	1.737 (0.429–7.031)	0.439				
Presence of glaucoma, yes	2.475 (1.030–5.952)	**0.043**	2.469 (0.465–13.101)	0.289	2.525 (0.169–13.585)	0.281
Mean IOP, mmHg	1.025 (0.986–1.066)	0.206				
Laterality, bilateral vs. unilateral	0.941 (0.472–1.876)	0.863				
Usage of glaucoma medications, yes	0.928 (0.467–1.842)	0.831				
Axial length, mm	1.912 (1.255–2.911)	**0.003**	2.311 (1.323–4.038)	**0.003**	2.213 (1.192–4.107)	**0.012**
History of intraocular surgery, yes	2.746 (1.348–5.594)	**0.005**				
History of trabeculectomy, yes	5.357 (2.248–12.764)	**< 0.001**	11.435 (2.748–47.577)	**< 0.001**		
History of cataract surgery, yes	0.702 (0.272–1.812)	0.464				
Presence of iridectomy, yes	6.657 (2.841–15.597)	**< 0.001**			11.113 (2.682–46.036)	**< 0.001**

The history of trabeculectomy and the presence of iridectomy were strongly associated with each other; thus, each was analysed separately in the multivariate analysis.

CI, Confidence interval; IOP, intraocular pressure; XFS, exfoliation syndrome.

Statistically significant values are shown in bold.

## Discussion

Although the pathogenesis of XFS is not fully understood, it is well known that XFS is an age-related, late-onset disease to which patients may be genetically predisposed and has associated environmental risk factors^1,3,5^. In this study, we compared the characteristics of early- and late-onset XFS and investigated the factors associated with early-onset XFS. Our results showed that eyes with early-onset XFS more frequently had a history of trabeculectomy or iris-damaging intraocular surgery and had a higher degree of axial myopia. These findings suggest that surgical trauma and myopia could be non-genetic risk factors for the development of XFS, especially for the early manifestation of the disease. To the best of our knowledge, this is the first study analysing the clinical aspects according to the onset of XFS in patients from a single ethnic group at a single institution.

Cases of early-onset XFS have been reported individually^14–16^. For example, a case of a 13-year-old girl with XFS who underwent ECCE was previously reported^17^. Additionally, a 17-year-old patient who underwent trabeculectomy was described as a case of early-onset XFS^16^. Another report presented four cases of XFS in patients younger than 50 years who had undergone multiple intraocular procedures^18^. Furthermore, a recent article systematically reviewed all the literature on early-onset XFS and discovered that all patients had an ocular history remarkable for intraocular surgery, ocular trauma, or ocular diseases, suggesting that the disruption of the blood-aqueous barrier (BAB) is a common triggering factor for early-onset XFS^10^. These report findings are consistent with our finding that a history of intraocular surgery at a young age was related to an increased risk of early-onset XFS.

In this study, the proportion of patients who underwent intraocular procedures before the manifestation of XFS was significantly higher in the early-onset group, which mainly included patients who underwent trabeculectomy. Indeed, all cases of XFS under the age of 52 years shared a history of trabeculectomy for the treatment of glaucoma, other than exfoliation glaucoma. These findings suggest an association between trabeculectomy and the early manifestation of XFS. We speculated that surgical iridectomy performed during trabeculectomy could trigger the early manifestation of XFM. In fact, in the early-onset group, two of three cases treated with intraocular surgery other than trabeculectomy had ECCE together with iridectomy. In contrast, no evidence of iridectomy was found in any of the 26 patients with a history of cataract surgery alone in the late-onset group. In addition, characteristic XFM was observed at the iridectomy site in an eye that had undergone trabeculectomy in the early-onset group (Fig. 1). These findings suggest that surgical procedures that traumatise the iris, such as trabeculectomy or iridectomy, may disrupt the BAB and produce several growth factors and cytokines that induce XFM formation.

The iris is rich in blood vessels, and tight junctions in the vascular endothelium of the iris are the main sites of the BAB^19^. The BAB can be compromised in various ways, such as by oxidative stress and ultraviolet exposure^19,20^. Once the BAB is disrupted, various proteins and the extracellular matrix in serum can accumulate in the AH, inducing abnormal production of XFM. These substances can create a vicious cycle by constantly damaging the BAB^8^. The expression of TGF-β1, a major regulator of extracellular matrix that induces the production of XFM, is elevated not only in the AH of eyes with XFS but also in the AH of eyes with juvenile idiopathic arthritis-associated anterior uveitis, wherein the BAB is broken^6,8,21^. When the iris is directly damaged by surgery or trauma as in the cases in this report, BAB destruction may be substantially greater, which is assumed to accelerate the early manifestation of XFM. Studies on the direct effect of iris damage on AH TGF-β1 levels remain limited; however, these studies may help clarify the possible link among surgical trauma, BAB disruption, and early manifestation of XFM.

In this study, long axial length was another factor associated with early-onset XFS, independent of a previous history of trabeculectomy or the presence of iridectomy. Although the mechanism is not clear, long axial length can be considered a predisposing factor for the development of early-onset XFS, while intraocular surgery serves as an environmental factor. Few case–control studies have reported the association between long axial length and XFS^9,11^, which makes it difficult to interpret our findings. This could be attributed to the fact that young patients with this condition have rarely been included in previous studies. Considering that myopic axial elongation occurs at a young age, myopia may have a greater effect on the development of early-onset XFS than on that of late-onset XFS. The causal relationship between the axial length and early-onset XFS should be validated in studies including a larger number of patients with early-onset XFS in the future.

Our findings showed that there was a marginal difference in the presence of glaucoma between the early-onset and late-onset groups. It is assumed that the higher prevalence of glaucoma is attributed to the higher proportion of trabeculectomy cases in the early-onset group. In fact, the presence of glaucoma was not significantly associated with early-onset XFS in the multivariable logistic regression analysis, while a history of trabeculectomy was associated. In addition, the presence of glaucoma is unlikely to affect the development of XFS, and rather vice versa. These findings suggest that a history of trabeculectomy was associated with the early-onset XFS irrespective of the presence of glaucoma.

This study has several limitations. First, as it was a retrospective study, we cannot be certain that early-onset XFS and its associated factors are causally linked. Second, there may be a gap between the actual onset of the disease and the detection of the disease. However, the majority of the patients were followed up regularly, and most of the patients who underwent surgery were also evaluated before surgery at our clinic, which could have minimised this gap. Third, although we found that a history of intraocular surgery involving iris trauma was associated with early-onset XFS, whether and how the timing of intraocular surgery affects the development of early-onset XFS remain unknown. Fourth, this study was conducted at a tertiary medical centre, and the high prevalence of concomitant glaucoma in XFS, both in the early-onset and late-onset groups, may have caused selection bias. In addition, those who had undergone ocular surgery at a younger age may be monitored more closely, which can affect the higher rates of detection of XFM. A population-based longitudinal study may be required to overcome this issue in the future. Finally, it is unknown how XFM, which occurs after trabeculectomy for glaucoma at an early age, affects the characteristics of the trabecular meshwork or the outcome of filtering surgery. This should be clarified in detail by further investigation to better understand the significance of early-onset XFS.

In conclusion, early-onset XFS was characterised by a higher prevalence of prior trabeculectomy and a higher degree of myopia than late-onset XFS. These results suggest that BAB disruption owing to surgical trauma may accelerate the production of XFM at a young age, especially in myopic eyes. Further studies are warranted to confirm these findings and determine their clinical relevance with a larger number of patients from various ethnic populations.

## Data Availability

The datasets analysed during the current study are available from the corresponding author on reasonable request.
